# Genetic diversity of macauba from natural populations of Brazil

**DOI:** 10.1186/s13104-015-1335-1

**Published:** 2015-09-04

**Authors:** Léo Duc Haa Carson Schwartzhaupt da Conceição, Rosemar Antoniassi, Nilton Tadeu Vilela Junqueira, Marcelo Fideles Braga, Adelia Ferreira de Faria-Machado, Joice Barbosa Rogério, Iara Duprat Duarte, Humberto Ribeiro Bizzo

**Affiliations:** Embrapa Cerrados, BR 020 km 18, Caixa Postal 08223, Brasília, DF 73310-970 Brazil; Embrapa Food Technology, Av. das Américas, Caixa Postal 29501, Rio de Janeiro, RJ 23020-470 Brazil

**Keywords:** *Acrocomiaaculeata*, Euclidean distance, Agroenergy, Genetic divergence

## Abstract

**Background:**

The macauba has been identified as the most promising native species for the production of vegetable oil and biomass. Several studies confirm its potential for numerous purposes (liquid and solid biofuels, food, cosmetics and pharmaceuticals), but this Brazilian biodiversity resource has been little explored, and work aimed at their domestication and genetic improvement are relatively recent. This study consisted of a multivariate approach to levels of trans fatty acids, oil yield and physical characteristics found in fruits of macauba of natural populations. The objective was to quantify the genetic variability among 35 genotypes of natural populations of macauba from 16 locations in different regions of Brazil. Euclidean Distance measurements were estimated and the cluster analysis obtained by the UPGMA method considering separately the fatty acid profile, and traits related to physical part and the fruits oil content.

**Results:**

It was observed the formation of seven groups for the profile of fatty acids and five groups for physical characteristics and oil yield. Large variations were observed for different types of mesocarp (pulp) fatty acids and kernel. Oleic acid (18: 1) in mesocarp was the largest contribution to the total divergence. The results indicate variations to the physical characteristics and oil yield, especially the oil percentage in mesocarp and weight of the whole fruit which contributed 64.58 % of the divergence between genotypes.

**Conclusions:**

The study identified genotypes potential to generate variability and obtaining selection gains, directing plant breeding programs according with demands of oils market.

## Background

The macauba (*Acrocomia aculeata* (Jacq.) Lodd. Ex Mart.) from the family Arecaceae, is a native palm tree widely distributed in Brazil, being abundant in the cerrado biome [1]. Several reports on its traditional use as a source of oil for the manufacture of soap and use in food, in addition to the use of leaves as forage for animal feed and crafts, endocarp to coal, fruits for human consumption and pulp flour for producing various food products [2–4]. In Paraguay, neighboring country of South America, the exploitation of fruit macaúba (*Acrocomiatotai*) has been practiced since 1940 [5], and in 2011 about 5000 tons of almond oil was produced and marketed [6]. Recently in Brazil, several studies have assessed the potential of macauba as a renewable biomass resource for generation of liquid and solid biofuels [7], as well as oil for food industry, cosmetics and pharmaceuticals [8].

The use of oil for various purposes depends on the fatty acid profile which is associated with nutritional value and physico-chemical characteristics of oil. Changes in expression of fatty acids in soybean have been observed due to environmental influences such as planting time and temperature [9, 10], or due to the stage of maturation of the grain [11, 12]. However, changes in fatty acid composition through conventional breeding are possible and have been successful in obtaining soybean to satisfy the market demand [13].

Despite its great potential as a source of oil besides several applications for its fruits, and diversity of uses considering the possible products and by-products obtained by oil extraction, the domestication and breeding program of macauba tree for oil yield has been incipient. Currently, new efforts are being focused on species with potential for biofuel production and it has been the main objective in projects funded for research involving this species. The production of biodiesel from macauba follows a technological approaches in the medium term, with the development of production systems of perennials tropical plants for oil production, which is not possible the cultivation of oil palm, because of environmental constraints [14]. In a recent review Dias [15] says that the contribution of improvement programs of specific plants for oil and biodiesel is small considering the annual growth of the Brazilian program of biodiesel production and placement of Brazil on the world stage.

According to Cruz [16] the success of a preservation or pre-improvement program is dependent on knowledge of the amount of variation in the species of interest. Studies of the genetic diversity in natural populations are important to quantify the variability, particularly in respect to traits of interest in addition to indicate collection sites and sampling strategies for the preservation and use of genetic resources. The macauba, even with the exponential growth of search actions in the last 10 years and the resulting increase in knowledge, still is a wild specie. Recently, several studies have been conducted in order to characterize their productive, morphological and biometric aspects [17, 18], phenology, reproductive [18–20] and molecular [21–23]. Those works were important in order to demonstrate the potential of this species in relation to the application of products and by-products and quantify the genetic diversity.

Genetic diversity studies in macauba related to fatty acid composition are non-existent and may be important to target improvement strategies to meet the needs of the oil market. Furthermore, this study also contributes to increasing knowledge about genetic diversity related to physical characteristics of fruit and oil yield among plants of macauba natural populations, in order to select genotypes for the maintenance of genetic variability and to obtain plants promising.

## Results and discussion

**Fig. 1:**
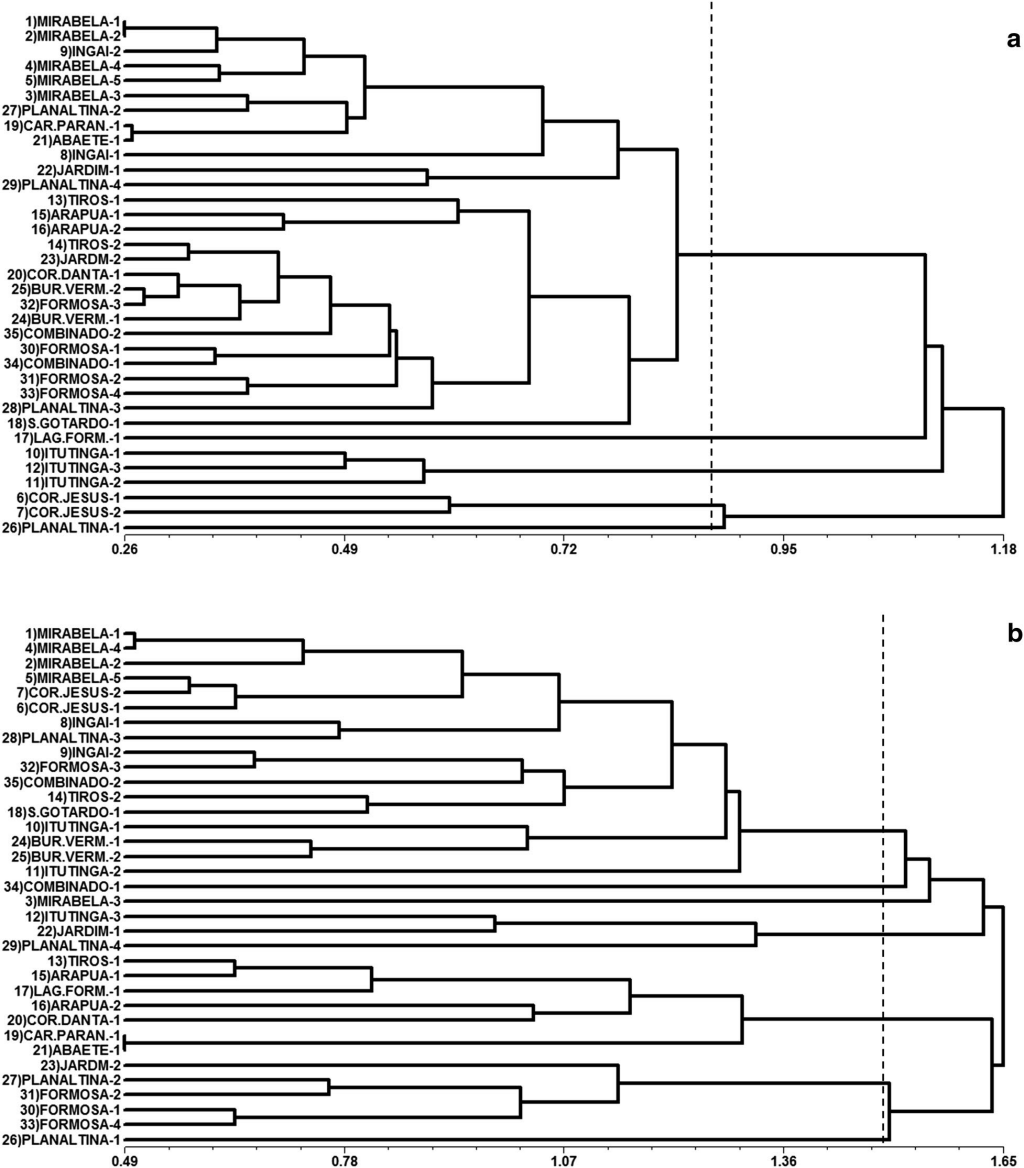
Dendrograms resulting from the analysis of 35 macauba genotypes, classified according to Euclidean Distance based on physical traits fruits and oil contente (**a**) and fatty acid profile of mesocarp and kernel oils (**b**), obtained by the UPGMA clustering method (Unweighted Pair-Group Average)

**Fig. 2:**
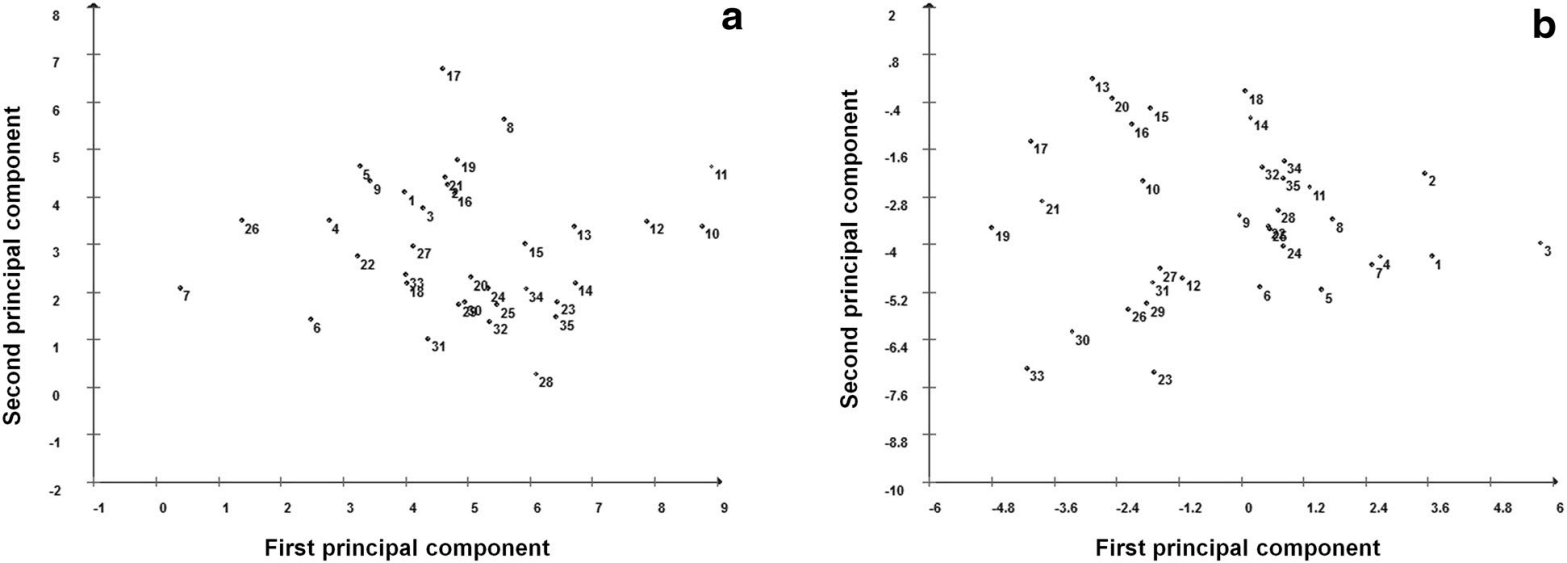
Two-dimensional graph of clustering of 35 macauba genotypes based on scores of the first and second principal components, considering physical traits fruits and oil contente (**a**) and fatty acid profile of mesocarp and kernel oils (**b**)

The dendrogram generated based on Euclidean distances presented the structuring of five groups among the genotypes considering the physical characteristics and oil content (Fig. 1a). The Itutinga genotypes formed a separate group, as well as the genotypes of the Coração de Jesus. Planaltina-1 and Lagoa Formosa-1 formed both isolated groups each. For the largest group (80 % genotype) observed a subdivision into two groups with greater proximity between Mirabela and Ingai, the state of Minas Gerais, and a second group of genotypes of the Alto Paranaíba region (Tiros-MG, Arapua-MG, Corrego Danta-MG and São Gotardo-MG), Buriti Vermelho-DF, Formosa-GO and Combinado-TO. Regarding dendrogram generated from the fatty acid profile (Fig. 1b) observed the structure formed into seven groups, with similarities when compared to analysis based on physical characteristics and oil content. Planaltina-1 again formed an isolated group. Mirabela-MG genotypes of the Montes Claros region, mostly, formed the largest group with the genotypes of Ingai, and totaled up yet genotypes of Coração de Jesus-MG, also in Montes Claros, and Buriti Vermelho-DF. Next to this larger group, two groups were formed by individual genotypes, Combinado-1 and Mirabela-3. Two other groups had the same trend to group genotypes with common origin. A group formed by the majority of genotypes Formosa-GO and another seven genotypes of the Alto Paranaiba region. The principal component analysis revealed that the dispersion of scores in cartesian axes, both the physical characteristics of the fruits and oil content (Fig. 2a) and to the fatty acid profile (Fig. 2b), showed the same pattern grouping and/or similarity between the 35 genotypes observed in dendograms. Other studies of genetic diversity in macauba found the same trend of generating clusters related to the genetic origin using both quantitative variables [24] as molecular data [22, 23].

There was the importance of the percentage of oil in the mesocarp character to differentiate between genotypes. This was the most relevant trait contributing 47.57 % to the total dissimilarity. The weight of whole fruit (17.1 %) also played an important role in quantified distances (Table 1). In a study of divergence held with babaçu, *Orbignyaphalerata*, the characters of greater contribution to discrimination of genotypes were the weight of kernels/plant, the weight of kernel/fruit weight and circumference at chest level [25]. The length of internodes, yield fruit/bunch and oil yield in the pulp were important to discriminate genotypes of tucuma, *Astrocaryumvulgare* [26]. Oleic fatty acid (18:1) and linoleic (18:2) in the pulp were the traits related to the profile of fatty acids which contributed most to the total divergence and differentiation of genotypes (Table 2) and explained 59.75 % of the total variation observed. The percentage of lauric acid (12:0) and oleic (18:1) kernel also had significant contribution (12.18 and 11.40 %, respectively).

**Table 1:** Estimates of relative contribution (Sj) of characteristics related to physical aspects of fruit and oil yield for genetic divergence among the genotypes macauba, based on Singh statistical [27]

Character	Sj	Relative contribution (%)
Whole fruit weight (g)	90,693.57	17.01
Epicarp (%)	30,834.67	5.78
Mesocarp (%)	56,563.09	10.61
Kernel (%)	3246.887	0.61
Endocarp (%)	24,114.24	4.52
Oil content of mesocarp—dry basis (%)	253,578.4	47.57
Oil content of kernel—dry basis (%)	50,968.33	9.56
Oil content of whole fruit—wet basis (%)	23,110.53	4.34

**Table 2:** Estimates of relative contribution (Sj) of characteristics related to the profile of fatty acids for genetic divergence among the genotypes macauba, based on Singh statistical [27]

Oil source	Character	Sj	Relative contribution (%)
Mesocarp	Lauric acid (C12 0)	4.9544	0.00
	Myristic acid (C14:0)	12.0266	0.01
	Palmitic acid (C16:0)	28,354.77	20.14
	Palmitoleic acid (C16:1)	3765.293	2.67
	Stearic acid (C18:0)	2104.355	1.49
	Oleic acid (C18:1)	55,773.67	39.61
	Linoleic acid (C18:2)	10,198.41	7.24
	Linolenic acid (C18:3)	136.5094	0.10
	Arachidic acid (C20:0)	5.9596	0.00
Kernel	Caprylic acid (C8:0)	853.1326	0.61
	Capric acid (C10:0)	807.3406	0.57
	Lauric acid (C12:0)	17,142.07	12.18
	Myristic acid (C14:0)	2121.19	1.51
	Palmitic acid (C16:0)	2069.523	1.47
	Stearic acid (C18:0)	466.7626	0.33
	Oleic acid (C18:1)	16,050	11.40
	Linoleic acid (C18:2)	928.4484	0.66
	Arachidic acid (C20:0)	3.0126	0.00

It was observed high levels of oil percentage for genotypes Itutinga-MG, Lagoa Formosa-MG and Tiros-MG, reaching values above 75 % oil in the mesocarp and 50 % in almond. While genotypes of Montes Claros region, Coração de Jesus, and Nucleo Rural Jardim-DF showed oil content in the mesocarp around 30 %, and the lower value found was in Planaltina-1, 14.65 % (Table 3). Positive and significant correlation was identified between PFI and MES, 0.56 (Table 4). Among the OMES and MES characters the correlation was significant but negative. That is, while bringing the mesocarp percentage relative to whole fruit, decreases its oil content. Similar results were found in other species of palm trees. In tucumã the total weight of the fruit was positively correlated with weight of mesocarp [28]. Ciconini et al. [4] found a positive association between fruit mass of macaúba and mass of mesocarp. In pupunha palm (*Bactrisgasipaes*), Santos et al. [29] observed a significant negative association between oil content and mesocarp percentage in the fruit. It Ciconini et al. [4] found no correlation between mass of flesh and oil content in the mesocarp. However, most association was found between oil content in the mesocarp (OMES) and oil yield the wet base (REND) 0.89, easily explained because the mesocarp of the fruit is the largest contributor to total oil (Table 4).

**Table 3:** Average values of weight of whole fruit (PFI) and percentage of epicarp (EPI), mesocarp (MES), kernel (AME) and endocarp (END) by weight of fruit, mesocarp oil content (dry basis) (OMES), kernel oil content (dry basis) (OAME) and oil yield (wet basis) (REND) observed in natural populations of macauba

No	Genotypes	PFI	EPI	MES	AME	END	OMES	OAME	REND
		g	%						
1	Mirabela-1	42.07	23.55	45.38	4.81	26.59	61.31	37.06	19.16
2	Mirabela-2	44.12	23.16	43.95	5.08	28.34	67.75	42.70	19.60
3	Mirabela-3	36.52	24.78	44.55	5.59	24.65	53.39	44.51	15.74
4	Mirabela-4	40.02	20.72	50.30	4.32	24.89	48.41	38.85	14.12
5	Mirabela-5	45.34	23.90	49.85	4.76	22.03	57.87	43.61	16.39
6	Cor.Jesus-1	49.58	22.72	46.83	6.90	28.79	30.00	36.82	6.58
7	Cor.Jesus-2	48.34	18.42	55.02	4.85	22.04	24.97	33.24	5.01
8	Ingai-1	44.82	35.01	36.06	5.30	23.16	74.28	39.97	20.68
9	Ingai-2	53.03	22.65	46.89	5.09	25.54	65.40	37.76	17.64
10	Itutinga-1	24.43	33.20	26.17	7.38	33.39	77.54	54.34	16.40
11	Itutinga-2	32.57	42.28	23.58	7.67	28.39	73.81	61.16	14.76
12	Itutinga-3	38.62	31.77	27.43	7.10	33.61	65.71	59.12	15.35
13	Tiros-1	27.72	22.69	38.86	6.03	32.43	76.56	50.59	17.85
14	Tiros-2	33.65	22.08	35.82	8.16	34.19	64.50	49.04	16.85
15	Arapua-1	43.68	19.70	40.76	7.77	32.04	65.42	52.27	18.91
16	Arapua-2	43.95	21.07	46.29	6.06	27.05	64.48	52.35	17.77
17	Lag. Formosa-1	51.36	23.73	51.22	4.95	20.66	77.43	56.81	25.21
18	S. Gotardo-1	58.62	18.82	43.43	8.65	29.36	43.77	51.04	12.52
19	Car. Paranaíba-1	53.38	27.00	41.60	5.55	26.27	64.47	51.60	17.02
20	Cor. Danta-1	37.91	21.91	40.38	6.86	31.20	49.90	46.76	13.89
21	Abaete-1	49.33	27.37	40.94	5.29	26.67	64.01	45.12	16.18
22	Jardim-1	34.60	26.47	44.44	5.61	22.81	32.79	43.26	8.25
23	Jardm-2	39.37	24.67	32.87	8.73	34.17	60.35	43.56	14.81
24	Bur. Vermelho-1	32.98	23.75	39.89	8.91	27.37	49.67	43.85	14.69
25	Bur. Vermelho-2	31.35	20.29	40.52	7.96	31.64	55.55	44.07	14.19
26	Planaltina-1	55.10	27.47	48.69	4.30	19.65	14.65	52.25	3.40
27	Planaltina-2	44.47	24.59	40.76	5.82	27.38	51.42	44.65	10.29
28	Planaltina-3	35.91	17.37	34.18	8.62	39.05	57.64	41.90	11.34
29	Planaltina-4	27.42	23.91	39.05	6.17	31.05	44.10	40.34	11.27
30	Formosa-1	48.92	19.76	40.26	9.23	30.79	54.55	45.26	13.46
31	Formosa-2	44.84	19.74	42.55	8.03	34.66	45.74	40.48	10.78
32	Formosa-3	38.93	20.87	37.84	8.43	33.16	50.41	42.40	13.58
33	Formosa-4	44.15	19.11	45.10	7.09	29.28	53.99	40.25	14.30
34	Combinado-1	42.49	22.35	38.26	10.39	29.53	56.38	46.45	17.46
35	Combinado-2	23.91	23.43	36.88	8.91	31.23	51.10	49.36	13.14
General average		41.24	24.01	41.05	6.75	28.66	55.70	45.79	14.53

**Table 4:** Pearson correlation between the physical traits fruits and oil content observed in 35 genotypes macauba

Character^a^	PFI	EPI	MES	AME	END	OMES	OAME	REND
PFI		−0.21^ns^	0.56**	−0.31^ns^	−0.47**	−0.24^ns^	−0.13^ns^	−0.05^ns^
EPI			−0.57**	−0.17^ns^	−0.20^ns^	0.35*	0.49**	0.16^ns^
MES				−0.56**	−0.65**	−0.48**	−0.50**	−0.16^ns^
AME					0.72**	0.06^ns^	0.19^ns^	−0.04^ns^
END						0.26^ns^	0.11^ns^	0.02^ns^
OL-MES							0.42*	0.89**
OL-AME								0.30^ns^
REND								

*ns* Not significant

**^,^* Significant at 1 and 5 % probability by t test

^a^weight of whole fruit (PFI) and percentage of epicarp (EPI), mesocarp (MES), kernel (AME) and endocarp (END) by weight of fruit, mesocarp oil content (dry basis) (OMES), kernel oil content (dry basis) (OAME) and oil yield (wet basis) (REND)

**Fig. 3:**
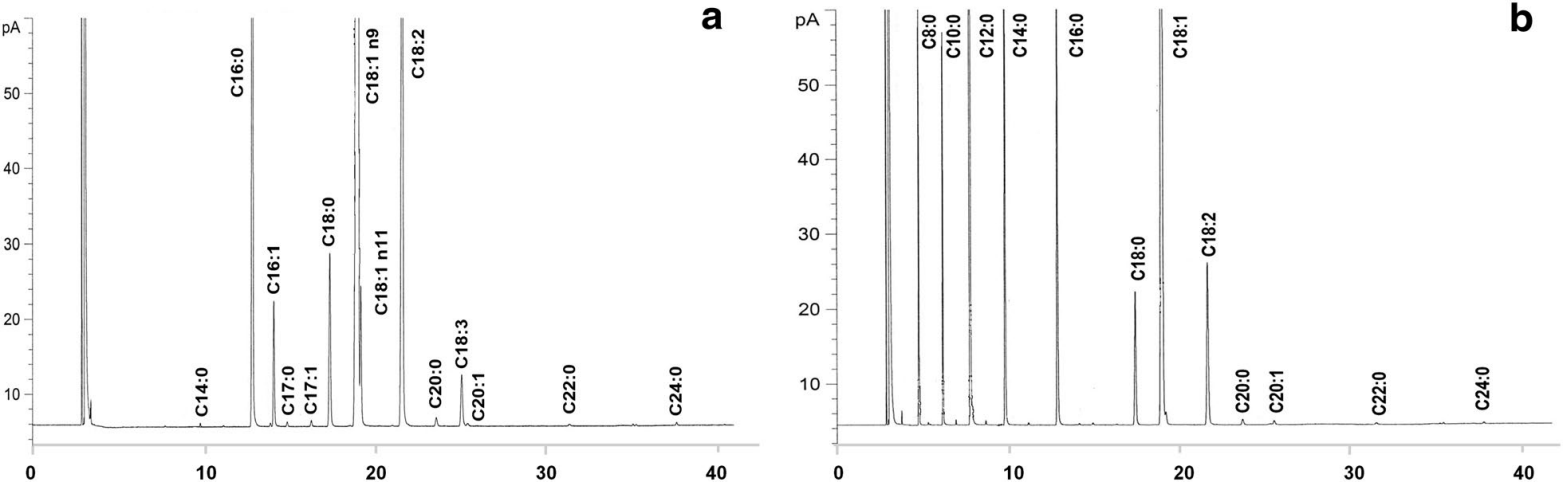
GC chromatograms of fatty acid methyl esters of mesocarp oil (**a**) and kernel oil (**b**) of macauba

Table 5 allows interpreting an interesting variation between the major fatty acids that comprise the mesocarp oil and almond Macauba (Fig. 3a, b). Genotypes Formosa-GO and Alto Paranaiba region, in Minas Gerais, showed relatively higher values for oleic fatty acid content (18:1), and generally lower for linoleic (18:2), especially Lagoa Formosa-MG with 79.12 and 10.22 % for oleic to linoleic acid. The highest oleic acid content and reduction of polyunsaturated fatty acids raise the oil stability by reducing or eliminating the need for hydrogenation [13]. The Planaltina-1 genotypes, Formosa-4 and Carmo do Paranaíba-1 showed the highest values for the lauric acid (12:0). Oils with high lauric acid content are valued in the international market for its wide use in the food industry and cosmetics [30]. The Mirabela-5 and Coração de Jesus-2 genotypes showed a peculiar characteristic of high linoleic acid content (18:2), omega-6, a polyunsaturated fatty acid that has properties beneficial to human health [31]. Genetic divergence studies based on the profile of fatty acids in palm trees as potential inaja (*Maximilianamaripa*), tucuma (*Astrocaruymvulgare*), babaçu (*Orbignyaphalerata*) among others, are rare and/or unavailable in the existing literature, including macauba. Some works with the reduced number of samples to bring specific information, for example, referring to babaçu oil composition [32] and tucuma [33]. These are important information about the species, but do not quantify the variability in populations or genotypes for fatty acid composition. Ciconini et al. [34] found variation in fatty acid profile of macauba genotypes for the central region of Mato Grasso and observed high content of unsaturated fatty acids in the mesocarp oil of macauba, due mainly to the high content of oleic acid (18:1).

**Table 5:** Average of the main fatty acids from mesocarp and kernel of fruits of macauba genotypes from natural populations

No	Genotypes	Fatty acid profile of mesocarp oil (%)			Fatty acid profile of kernel oil (%)	
		Palmitic (C16:0)	Oleic (C18:1)	Linoleic (C18:2)	Lauric (C12:0)	Oleic (C18:1)
1	Mirabela-1	22.30	54.90	15.22	34.33	29.47
2	Mirabela-2	20.83	55.06	15.20	31.89	32.55
3	Mirabela-3	26.61	48.16	12.60	30.67	30.78
4	Mirabela-4	23.41	55.16	13.62	35.49	30.02
5	Mirabela-5	19.10	53.64	19.71	38.68	26.38
6	Cor.Jesus-1	18.15	58.97	16.68	40.27	25.41
7	Cor.Jesus-2	20.04	53.16	19.37	36.88	28.65
8	Ingai-1	19.50	55.03	14.31	36.85	30.49
9	Ingai-2	14.79	61.80	16.53	36.16	32.03
10	Itutinga-1	15.87	65.98	8.44	40.21	26.97
11	Itutinga-2	19.29	61.01	9.58	35.06	31.97
12	Itutinga-3	20.20	56.85	10.53	39.37	28.52
13	Tiros-1	7.96	73.32	15.40	37.72	33.02
14	Tiros-2	14.64	64.44	14.40	36.22	32.93
15	Arapua-1	8.05	75.52	13.12	38.26	31.62
16	Arapua-2	14.07	62.14	18.39	39.17	30.02
17	Lag. Formosa-1	6.93	79.12	10.22	40.81	28.96
18	S. Gotardo-1	10.93	68.96	13.80	35.72	33.21
19	Car. Paranaíba-1	12.91	68.88	12.52	45.31	23.61
20	Cor. Danta-1	11.49	68.86	13.99	37.98	30.82
21	Abaete-1	12.45	69.05	12.77	42.57	26.99
22	Jardim-1	22.81	56.31	13.53	34.94	31.88
23	Jardm-2	23.33	55.14	14.55	40.35	24.58
24	Bur. Vermelho-1	20.28	63.45	10.59	36.20	27.90
25	Bur. Vermelho-2	22.81	59.52	8.81	38.21	28.09
26	Planaltina-1	13.49	60.42	17.61	46.98	20.37
27	Planaltina-2	14.90	62.10	17.50	41.93	25.85
28	Planaltina-3	18.48	60.57	15.70	35.34	30.67
29	Planaltina-4	19.31	58.44	14.56	40.52	25.11
30	Formosa-1	12.13	67.16	13.30	41.78	23.63
31	Formosa-2	12.73	66.98	13.44	40.80	24.13
32	Formosa-3	13.36	64.41	17.17	33.99	32.46
33	Formosa-4	14.93	65.99	11.21	44.54	19.78
34	Combinado-1	18.40	64.02	10.26	31.63	33.00
35	Combinado-2	19.69	57.22	16.90	34.73	32.04
General average		16.75	62.05	14.04	38.04	28.68

## Conclusions

Genetic variability is significant between genotypes macauba, and interesting sites collections were aimed at conservation of plant genetic resources to establish a breeding program. Genotypes of Alto Paranaiba region (Lagoa Formosa and Tiros), Lavras region (Itutinga and Ingai) and Montes Claros region (Mirabela) are promising for generating populations (intra or inter progenies crossed) with prospects of obtaining genotypes upper and significant gains in selection for oil yield. Improved populations derived from crosses between progeny of genetically distant genotypes and superior performances are promising for selection.

The variability is high in chemical composition from oils, related to fatty acid levels found in the pulp and almond 35 characterized genotypes. It is possible the breeding of this species for different focuses, meeting diverse demands of the fatty acids market for the variation found in the oil composition of both the mesocarp (pulp) and kernel.

## Methods

Natural populations of macauba (*Acrocomiaaculeate*) from six regions in Brazil were assessed: Regions of Montes Claros, Alto Parnaíba and Lavras in Minas Gerais State, region of Formosa, Goiás State, Combinado in Tocantins State and Distrito Federal (Table 6).

**Table 6:** Populations, collecting sites macauba fruit samples and genotypes/local

Region–population	Location of collection	Genotypes/local
1. Region of Montes Claros-MG	Mirabela-MG	1, 2, 3, 4 e 5
	Coração de Jesus-MG	6 e 7
2. Region of Lavras-MG	Ingaí-MG	8 e 9
	Itutinga-MG	10, 11 e 12
3. Region of Alto Paranaíba-MG	Tiros-MG	13 e 14
	Arapuá-MG	15 e 16
	Lagoa Formosa-MG	17
	São Gotardo-MG	18
	Carmo do Paranaíba-MG	19
	Córrego Danta-MG	20
	Abaeté-MG	21
4. Distrito Federal	Núcleo Rural Jardim-DF	22 e 23
	Núcleo Rural Buriti Vermelho-DF	24 e 25
	Planaltina-DF	26, 27, 28 e 29
5. Formosa-GO	Formosa-GO	30, 31, 32 e 33
6. Combinado-TO	Combinado-TO	34 e 35

The fruits of macauba were collected from mature bunches, frozen and sent to Fats and Oils Laboratory (Embrapa Food Technology) for physico-chemical analysis. All parts of the fruit were weighted. Physical traits observed were: weight of whole fruit (PFI), percentage of weight of epicarp (EPI), mesocarp (MES), kernel (AME) and endocarp (END) by weight of the fruit; and oil production traits: mesocarp oil content in dry basis (OMES), kernel oil content in dry basis (OAME) and oil yield (wet basis) (REND). The epicarp was removed, the mesocarp was cut and lyophilized, the woody endocarp was broken and the kernel was dried in an air-circulating oven at 60 °C. The oil extraction was performed in triplicate on a Soxhlet apparatus for 16 h using petroleum ether (bp 30–60 °C) as solvent. The fatty acid methyl esters were prepared according to Hartman and Lago [35] in triplicate. In brief: the saponification step of oil was carried out with potassium hydroxide (0.5 M) in methanol at 70 °C for 4 min with occasional agitation and the methylation step with HCL in methanol was accomplished during 3 min at 70 °C. The FAME were obtained by addition of ethyl ether followed by water washing, drying with anhydrous sodium sulfate and dilution with dichloromethane. Gas chromatography was performed in a Agilent 6890 chromatograph fitted with a cyanopropylsiloxane capillary column Quadrex 007 (60 m × 0.32 mm × 0.25 µm), and the following conditions: initial column temperature was set at 100 °C and held for 3 min, increased to 150 °C at 50 °C/min, further increased to 180 °C at 1 °C/min and finally increased to 200 °C at 25 °C/min and held for 10 min. Carrier gas used was hydrogen, at 1.4 mL/min (measured at 100 °C). Injection of 1.0 µL of a 2 % dichloromethane solution of the sample was done in an injector operating at 250 °C and split mode (1:50) and FID detector was kept at 280 °C. Results were expressed as weight percent (area normalization). Identification of FAME was based on comparison of retention times with those of NU CHEK standards 62, 79 and 87 (Elysian, MN).

For statistical analysis were considered two groups of characters: (1) fatty acid profile; and (2) physical characteristics of fruit and oil yield. The experimental units were constituted of 3–6 fruits samples, and the average value obtained for each character used as observed data. Multivariate statistical procedures were performed as follow: (1) estimation of genetic distances between genotypes from Euclidean distances calculated based on the characters evaluated; (2) the relative contribution of the variables were estimated for the total divergence [27]; (3) cluster genotypes by hierarchical method UPGMA (Unweighted Pair-Group Average); (4) principal component analysis. Estimates of phenotypic correlations by the method of Pearson also were carried to verify the degree of association between the physical characteristics and oil yield. The analyzes were performed using the Genes software [36] and NTSYS pc 2.1 [37].
